# Supplementary material for: Socio-community care of people with disabilities: Experiences of caregivers living in south-central zone of Chile
Source: PLoS One. 2026 Jan 21;21(1):e0339403. doi: 10.1371/journal.pone.0339403 (PMC12822954; doi:10.1371/journal.pone.0339403)
Supplement: S1 Appendix — (DOCX) [file pone.0339403.s001.docx]

**Appendix 1**

**Interview Guide Caregiver**

Participant’s Name:

Date:

Place of Interview:

**Personal Dimension of the Relationship with Care Tasks**

• How is your week going?

• How did you come to do care work or caregiving tasks?

• What care tasks do you currently perform or have performed in the past? How do you feel when doing them or when you did them? Could you give me another example?

• How much time do you spend performing care tasks?

• What is your relationship with the person you care for?

• How do you think the person you care for perceives or receives your care work?

• How do you take care of yourself? Or what makes you feel cared for?

## Socio-Community Dimension of Care

• Do you have networks or connections with people or institutions that support care tasks?

• How is your relationship with your neighborhood or local territory?

• Do you feel supported by your community in your caregiving work?

• How do you imagine the territory or community should be organized to support caregiving tasks?

## Political Dimension of Care

• What do you think the role of the Chilean State should be in care or personal assistance tasks?

• Do you consider that care work should be recognized as a form of labor?

• How do you view the work of SENADIS (National Disability Service) in relation to care tasks?
