# Supplementary material for: Socio-community care of people with disabilities: Experiences of caregivers living in south-central zone of Chile
Source: PLoS One. 2026 Jan 21;21(1):e0339403. doi: 10.1371/journal.pone.0339403 (PMC12822954; doi:10.1371/journal.pone.0339403)
Supplement: Attachment — Submitted filename: Review of Socio-community care of people with disabilities.docx [file pone.0339403.s002.docx]

Review of “Socio-community care of people with disabilities: Experiences of caregivers living in south-central Zone of Chile”

Comments to the author:

The article proposes to analyze the perceptions of caregivers regarding the construction of socio community care for people with disabilities in the south-central Zone of Chile, using a qualitative approach from a situated feminist perspective. Both the justification for the importance of the study and the objectives are clear. In addition, the article is presented in an intelligible fashion and is written in standard English.

My main criticism is that the study is decontextualized from the original theoretical discussion about unpaid care labor – in the context of the so-called "Care Economics" – which began in the 1990s in the United States. See, for example, some pioneering works in the area such as:

England, Paula and Folbre, Nancy. 1998. “The Cost of Caring.” The Annals of the American Academy of Political and Social Science 561, 39-51

Himmelweit, Susan. 1996. “Conceptualising Caring”. Paper presented at the International Association for Feminist Economics Conference, Washington D.C., July 1996

Nelson, Julie A. 1999. “Of Markets and Martyrs: Is it OK to Pay Well for Care?” Feminist Economics 5: 3, 1-17

In the Discussion section (pp. 14-16), a preliminary and cursory literature review on the topic of care, particularly in the context of caring for disabled individuals, is presented. I propose a reorganization of the article to include a separate section for contextualization and a comprehensive literature review on unpaid care labor immediately following the introduction. This structural adjustment will facilitate a more coherent analysis of the research findings in relation to existing theoretical studies.
