# Supplementary material for: Socio-community care of people with disabilities: Experiences of caregivers living in south-central zone of Chile
Source: PLoS One. 2026 Jan 21;21(1):e0339403. doi: 10.1371/journal.pone.0339403 (PMC12822954; doi:10.1371/journal.pone.0339403)
Supplement: Attachment — Submitted filename: Authors Responses. Plos One.docx [file pone.0339403.s004.docx]

| **Editor’s Comments** | **The authors' responses are highlighted in yellow in the manuscript.** |
| --- | --- |
| Thank you for stating the following financial disclosure:  "This research was financed by ANID-Fondecyt Postdoctoral N°3220665."  Please state what role the funders took in the study.  If the funders had no role, please state: "The funders had no role in study design, data collection and analysis, decision to publish, or preparation of the manuscript.""  If this statement is not correct you must amend it as needed.  Please include this amended Role of Funder statement in your cover letter; we will change the online submission form on your behalf. | Statement embedded in the text, page 19. |
| In the online submission form, you indicated that "Data are available upon request as the study has not been completed. These can be requested from the principal investigator, first author of this article."  All PLOS journals now require all data underlying the findings described in their manuscript to be freely available to other researchers, either 1. In a public repository, 2. Within the manuscript itself, or 3. Uploaded as supplementary information.  This policy applies to all data except where public deposition would breach compliance with the protocol approved by your research ethics board. If your data cannot be made publicly available for ethical or legal reasons (e.g., public availability would compromise patient privacy), please explain your reasons on resubmission and your exemption request will be escalated for approval. | All the necessary information regarding the participants and their experiences in caring for people with disabilities is in the manuscript. |
| Please include your full ethics statement in the ‘Methods’ section of your manuscript file. In your statement, please include the full name of the IRB or ethics committee who approved or waived your study, as well as whether or not you obtained informed written or verbal consent. If consent was waived for your study, please include this information in your statement as well. | The requested information is included on page 8, in the 'ethical considerations' section. |
| **Reviewer 1** | **Authors’ response** |
| My main criticism is that the study is decontextualized from the original theoretical discussion about unpaid care labor – in the context of the so-called "Care Economics" – which began in the 1990s in the United States. See, for example, some pioneering works in the area such as: England, Paula and Folbre, Nancy. 1998. “The Cost of Caring.” The Annals of the American Academy of Political and Social Science 561, 39-51 Himmelweit, Susan. 1996. “Conceptualising Caring”. Paper presented at the International Association for Feminist Economics Conference, Washington D.C., July 1996. Nelson, Julie A. 1999. “Of Markets and Martyrs: Is it OK to Pay Well for Care?” Feminist Economics 5: 3, 1-17 | The conceptual and theoretical framework has been strengthened. The suggested literature has been incorporated into the manuscript. See pages 4 and 5; 16 and 17. |
| In the Discussion section (pp. 14-16), a preliminary and cursory literature review on the topic of care, particularly in the context of caring for disabled individuals, is presented. I propose a reorganization of the article to include a separate section for contextualization and a comprehensive literature review on unpaid care labor immediately following the introduction. This structural adjustment will facilitate a more coherent analysis of the research findings in relation to existing theoretical studies. | The introduction has been restructured according to the suggestions. The discussion has been strengthened with other studies in the field. See pages 4 and 5; 16, 17, and 18. |
| **Reviewer 2** | **Authors’ response** |
| I suggest detailing the methodology more clearly, what design was used. In addition, I suggest organizing the methodology sections in a more logical way. | The suggestion has been incorporated. See pages 6, 7, 8, and 9. |
| The “context of the study” section seems unrelated to the rest. I suggest making the link. The same with table 1, where the column (% of rural population) seems more like an isolated background, unless it is integrated into the story. Basically, answering the question: why are both pieces of information important? | The 'Study Context' section is explained more clearly. The rurality background has been removed from Table 1. See pages 6 and 7. |
| Does the theoretical approach chosen for the analysis have any contribution other than separating institutional and community care? This division is quite classic when carrying out support analysis, so I wonder if the author and theoretical approach chosen could contribute something other than that division. | The conceptual and theoretical framework has been better argued and structured. See pages 3, 4, and 5. |
| Regarding the first part of the results, I suggest explaining in the methodology section how the internal categories were established for the two predetermined categories. | The suggestion has been incorporated. See the 'Data Analysis' section on page 9. |
| Regarding the results, very general statements are made that would benefit from integrating more data or examples. An example is the allusion to the fact that care in the family is feminized and ableist. However, there is no detailed discussion with data on what this means and how it is observed. In most of the categories, this format is replicated, which makes the depth of the data and the richness of the results as a contribution disappear. | The suggestion has been incorporated, deepening the participants' experiences and their analysis in the results. See pages 9 to 16. |
| I consider that the discussion requires some attention. Although its content is relevant to the topic, it is presented more as background than as a discussion of the findings. Some sections can be written as an introduction, without the results. I suggest going into more depth so that the findings are clearly reflected. | The suggestion has been incorporated. The discussion has been expanded by including other studies in the field. See pages 16, 17, and 18. |
| In the conclusions, it is established that the integration of both networks reduces institutionalization. This seems to be new information that has not been presented or analyzed before, and due to the characteristics of a conclusion, I consider that it should not be presented in a new and general way there. It is an interesting dimension that should be addressed in the discussion, if the findings lead in that direction. | This element has been included in the following sections: Introduction, Results, and Discussion. |
| In general, rurality does not seem to be relevant to the study, despite the fact that it is presented in the title and as an inclusion criterion. There is nothing on this aspect even in the bibliography, and it seems that it was not treated as a relevant topic in the interview questions. Was it? If this was relevant to the analysis, I suggest incorporating it. If not, I suggest considering it. | The suggestion has been incorporated throughout the text. |
