# Supplementary material for: Socio-community care of people with disabilities: Experiences of caregivers living in south-central zone of Chile
Source: PLoS One. 2026 Jan 21;21(1):e0339403. doi: 10.1371/journal.pone.0339403 (PMC12822954; doi:10.1371/journal.pone.0339403)
Supplement: Attachment — Submitted filename: Response to Reviewers_October_2025.docx [file pone.0339403.s005.docx]

| **Reviewers’ Suggestions** | **All suggested revisions have been incorporated into the document and are highlighted in yellow colour for clarity.** |
| --- | --- |
| **Reviewer #1:** Re-reading the text after the review, it occurred to me that the details provided by the methodology may not be sufficient to allow the research to be reproduced elsewhere. Therefore, I will also suggest the authors to publish the questionnaire used in the survey as an appendix. | The interview guide used with the participants is included as supplementary material in the Appendices, following the list of references. |
| **Reviewer #2:** Most of the comments have been appropriately addressed. However, the relationship between results and discussion remains weak and needs to be strengthened. The statement regarding rurality remains incomplete. Additionally, the data are not available at the indicated DOI. | We have strengthened the connection between the results and the discussion by explicitly highlighting how the findings inform and extend the main theoretical and empirical debates addressed in the introduction.  After careful consideration, we decided to remove this aspect from the manuscript, as it was not central to the study’s main focus. This change contributes to greater conceptual coherence and clarity in the argument.  The DOI link has been verified and corrected. |
